# Probing the mystery of Chinese medicine meridian channels with special emphasis on the connective tissue interstitial fluid system, mechanotransduction, cells durotaxis and mast cell degranulation

**DOI:** 10.1186/1749-8546-4-10

**Published:** 2009-05-29

**Authors:** Peter Chin Wan Fung

**Affiliations:** 1 Department of Medicine, Department of Electrical and Electronic Engineering, University of Hong Kong, Hong Kong, PR China

## Abstract

This article hypothesizes that the Chinese medicine meridian system is a special channel network comprising of skin with abundant nerves and nociceptive receptors of various types, and deeper connective tissues inside the body with the flowing interstitial fluid system. These meridian channels provide efficient migratory tracks mainly due to durotaxis (also including chemotaxis) for mast cells, fibroblasts and other cells to migrate and carry out a number of physiological functions. Acupuncture acting on meridian channel causes cytoskeletal remodeling through mechanotransduction, leading to regulation of gene expression and the subsequent production of related proteins. Also, stimulation on cell surface can trigger Ca^2+ ^activities, resulting in a cascade of intra- and inter-cellular signaling. Moreover, nerve endings in the meridian channels interact with mast cells and induce the degranulation of these cells, leading to the release of many specific biomolecules needed for homeostasis, immune surveillance, wound healing and tissue repair. Acupoint along a meridian channel is a functional site to trigger the above functions with specificity and high efficiency.

## Introduction

Acupuncture, a major component in Chinese medicine, has a history of well over two thousand years and is effective to maintain good health and to treat various diseases [1]. According to classic acupuncture theory, there is a network of meridian channels inside the human body with acupoints on the skin and deeper tissues. Needling at the acupoints modulates the physiology of the body through the meridian channel network. The anatomical structures and physiological functions of the Chinese medicine acupoints, meridian channels and acupuncture have not been shown to have equivalents in modern biomedical science. Despite enormous research endeavors especially in China, there is still no consensus about (a) the anatomy of the Chinese medicine meridian channels and acupoints; (b) the physiology of acupuncture and moxibustion; (c) the scientific explanation, in modern physio-pathological terms, of the beneficial effects of acupuncture and moxibustion.

Some research findings suggested that the anatomical structure of meridian channels and acupoints are related to the connective tissues and the connective tissue interstitial fluid (CTIF) system [2-9]. Based on interdisciplinary analysis of certain characteristics of the CTIF system, and integration of advances in Chinese medicine and biomedical science research, a new hypothesis for the meridian structure plus acupuncture physiology is proposed in this article. The hypothesis, referred as the CFMDD hypothesis, defines the meridian structure embedded in the Connective Tissue Interstitial Fluid system with acupuncture in action explained by Mechanotransduction, cells Durotaxis and mast cell Degranulation.

The objective of this article is to explain this integrated picture of meridian channel structure and acupuncture in action at acupoints based on modern concepts of biomedical science.

### The Connective Tissue Interstitial Fluid (CTIF) system

Among several primary tissues in humans, connective tissue is the most abundant and widely distributed one. Connective tissue consists of collagen fibers, proteins such as elastin, fibronectin, laminin and proteoglycans. The proteoglycans which form extremely thin fibrils interact with the surrounding interstitial fluid to form a gel-like environment [10]. The CTIF system forms a body fascia frame with connected layers [9,11] which embeds the neurovascular tracts and connects tunicae around the visceral organs. The frame also extends to form the periosteum which supplies vascular structure to the bone, which can be considered as just another connective tissue or extracellular matrix [12]. Thus the CTIF system with the "hard part" and "soft part" is an integrated network to maintain the integrity of body shape against gravity.

Electrolytes, proteins, O_2_, CO_2_, pass through this connective tissue-interstitial fluid (CTIF) system in transit. The cells in the CTIF system include macrophages, lymphocytes, T- and NK cells, eosinophils, adipocytes, plasma cells, fibroblasts, chondroblasts, osteoblasts, stem cells and mast cells.

### Special properties of the CTIF system and the Chinese medicine meridian channels

The interstitial fluid (IF) transports nutrient proteins, electrolytes, oxygen and water from the blood circulation to tissues of the whole body for their functions. The IF also sends CO_2 _and cellular excreta from these tissues back to the circulation and lymphatic system respectively. The nerve endings of various types of nerves are rich in the skin. The superficial fascia of the CTIF system therefore embeds a large numbers of nerve endings which contain specific and polymodal nociceptive receptors that carry out the three functions: (a) nociceptive signal as bio-warning; (b) facilitating reflexive modulation of organs; (c) defense mechanism via immune effector functions [13-17].

Mechanical, thermal, chemical or polymodal receptors are abundant in the CTIF system [17]. Moreover, there is an abundance of nociceptive receptors of various types in the deep fascia layers which are close or connecting to neurovasculature as well as lymphatic structure. For examples, the inter-muscular septa, tunicae around visceral organs, periosteum around bones are such deeper fascia layers [9-11]. According to Chinese medicine, the meridian channels support and regulate the functions of visceral and other organs. There is accumulative evidence that most, if not all, of the earth positions (the deepest position of the three acupuncture needling positions of depth, i.e. "sky", "man", "earth" positions) [18] of the acupoints are located in the connective tissues of the CTIF system [2-9]. Thus, the author hypothesizes that those channels which can play the strongest supportive roles to organs form the Chinese medicine meridian channels.

### What are acupoints?

High density sites of polymodal and specific nociceptive receptors near neurovascular structure, lymphatic vessel, mast cells become acupoints. Acupuncture applied at the collagen fibers embedding nerve fibers (Aδ and C) could send signals to the nerve fibers via the fundamental mechanotransduction mechanism [19]. At a dense neurovascular structure which also innervates the lymphatic vessel, acupuncture at one branch of a "sensory tree" could therefore affect the blood circulation and lymphatic pumping activities [20].

Since nerve fibers are connected to one or more spinal cord segments, the stated mechanical stimulation can be transmitted to internal organs via the somato-visceral organ reflex [21-23]. Thus many sites with high density of polymodal and specific nociceptive receptors could be sites fulfilling part of the function of acupoints [24].

However, a polymodal (or certain specific) nociceptive receptor can also carry out certain effector functions [13-17]. With secretion of small amounts of neuropeptides like substance P and calcitonin-gene-related-peptide (CGRP) from nerve endings arising from noxious stimulus, the effector functions that can be fulfilled are very limited. Those polymodal and other specific receptors that are near mast cell migration tracks would have much better chances of interaction with the mast cells, leading to the degranulation of these "storage cells". The released biochemicals could engage in at least 12 physio-pathological functions under the general areas of homeostasis, immunity responses, repair and growth [25-27]. These functions can be grouped together to be called effector functions, as if controlled by efferent nerves from the brain. Moreover, the biochemicals involved in the effector functions can diffuse readily in a flowing interstitial fluid.

Thus as an outcome of biological development, sites with high density of polymodal and specific receptors which are also near fine blood, nervous, lymphatic structures, as well as near mast cells would develop into functional sites with high efficiency. Maneuvering with needles at these sites, which are the Chinese medicine acupoints, could lead to therapeutic effects. In fact, acupoints have been found to be near dense neurovascular structure, lymphatic structure via anatomical analysis [7], and the mast cell densities were found to be high around acupoints [28-32].

### The relation between acupuncture and mechanotransduction

Biophysical forces (such as mechanical and electrical forces) acting on the cell surface are effective and fast, leading to intracellular and intercellular architecture remodeling and resulting in the occurrence of accompanying biochemical reactions [19]. It was shown that the nucleus, focal adhesion complex, the extracellular matrix (ECM) in connective tissue and gap junction form an integrated network to transmit mechanical stimuli, resulting even in gene regulation [33-35]. The connective tissues provide a living architecture for mechanical transduction which leads to subsequent biochemical responses in complex living organisms.

Acupuncture applied to connective tissue causes cytoskeletal remodeling of mechanically connected cells. A pull by the connective tissue to the mechanically connected distal cells would apply stress to the surface integrin receptors. There is growth of focal adhesion, leading to a stress-dependent increase in cytoskeletal (CSK) stiffness. The CSK stiffness changes because there is rearrangement of the microfilaments (MF), microtubules (MT) and intermediate filaments (IF). Since these structures join directly or indirectly to the nucleus surface (after such CSK remodeling), expressions of different genes are affected to produce the relevant proteins to maintain the cell integrity and the necessary physiological processes in response to the stimulus. Thus, cell nucleus, via mechanotransduction, can react directly to mechanical stress (which is initiated by acupuncture) at the cell surface receptor. Such mechanical stress when applies at the cell surface also triggers intracellular Ca^2+ ^oscillation and intercellular Ca^2+ ^wave, leading to a cascade of biochemical events. Details will be ready for publication shortly.

According to the electron-microscopic work of Messlinger [20], an example of Aδ nerve trunk has three branches. One branch innervated the neighbouring venous vessel, another branch innervated the lymphatic vessel, whereas the third branch was embedded in collagen fibers forming the "sensory tree". The main trunk is within the perineurium but with bead-like structure exposing in part of the main branch and all the three branches stated. The Schwann cell that myelinated the main trunk of the sensory tree was actually detached from the axon so that more bead-like structure could be exposed by mechanical pulling of the collagen fibers (like acupuncture action) which embed the third nerve branch. The beads contain more mitochondria and other vesicles which are capable of releasing neurotransmitters. Mechanical maneuvering of the collagen fibers would transmit mechanical signals to the connected blood and lymphatic vessels through the mechanotransduction mechanism. There are abundant Aδ fiber and C fiber endings in the CTIF system. Thus acupuncture could influence circulation property and lymphatic activity, as well as sensitization of polymodal receptors by inducing change in collagen tension.

### Formation of favorable tracks of cell migration in the CTIF system due mainly to durotaxis

Cells have long been known to orient and migrate responding to gradients of various potentials like photo-, galvano-, geo-, chemo-, hapto-taxis [36-39]. Another cell migration process relevant to acupuncture research is durotaxis. Durotaxis is a process via which cells migration is guided by gradients of substrate rigidity. Mechanical properties of ECM have been reported to affect fibronectin fibril assembly inside the cell [40], to change cytoskeletal stiffness [34], strength of integrin-cytoskeleton linkages [41,42]. These factors not only affect the cell structure, but also cell locomotion. It was demonstrated [43] that when cells are cultured on substrates of different rigidities (but with the same chemical properties), the morphology and motility rates of cells are different. Lo *et al*. [42] demonstrated 3T3 fibroblasts migrate into the rigid substrate. In a more recent study, Guo *et al*. [44] also showed that migration of 3T3 fibroblasts could be directed by altering the tension of the substrate. Similar process is likely to occur in acupuncture which directs cells migration by affecting the stiffness of the pathway in the CTIF system.

### Special migration tracks for fibroblasts and mast cells in the CTIF system are correlated with meridian channels

There are many "loose cells" residing in connective tissues including macrophages, lymphocytes, adipocytes, plasma cells, eosinophils, fibroblasts, chondroblasts, osteoblasts, stem cells and mast cells [45]. The solid substrate tissues can serve as the tracks of migration for the cells for physiological functions. The author hypothesizes that there are special migration tracks for fibroblasts and mast cells. Fibroblasts sustain collagen fibers [10] and mast cells upkeep the proliferation of fibroblasts [27] which tend to migrate along collagen fibers with higher stiffness [42,44]. The anatomical findings of Yuan *et al*. [9] suggest that the densities of the collagen fibers plus some proteins are higher along certain tracks correlating with the Chinese medicine meridian channels. On the other hand, mast cell densities were found to be higher around acupoints than nearby non-acupoints [28-32] and acupoints are along the meridian channels. These four sets of experimental findings suggest the formation of special migratory tracks for fibroblasts and mast cells in the CTIF system correlating with Chinese medicine meridian channels.

### Mast cells degranulation caused by acupuncture

Mast cells (size ~10–20 μm) are produced in bone marrow and migrate to the blood stream, peripheral tissues, and eventually to various types of connective tissues, adjacent to blood and lymphatic vessels and to the sites associated with peripheral nerves [25,46-48]. Mast cells can go through multiple cycles of de- and re-granulation for regulating the release of at least 15 types of biomolecules including serotonin, proteases, heparin, granulocyte macrophage colony-stimulating factor (GM-CSF), leukotrienes, interleukins, tumor necrosis factor-α (TNFα), calcitonin, nerve growth factor (NGF), stem cell factor (SCF), substance P, histamine, prostaglandin, thromboxane, and other peptides like fibroblast growth factor (FGF) [25-27]. These biomolecules, working separately or cooperatively, are involved in (1) allergy response, (2) acquired immunity, (3) innate immunity, (4) maintaining the life of sensory neurons, (5) inflammation, (6) supporting the growth of T cells and various tissues, (7) metabolic rate, (8) noxious stimuli response, (9) blood vessel tone regulation, (10) fibroblast growth, (11) wound healing, and (12) osteoblast formation [27]. Mast cells are believed to interact with connective tissue matrix components through integrins [49]. There is ample evidence of mast cell – nerve cell interaction [50-52]. The interaction between mast cells and nerve cells would cause degranulation of the former leading to the release of said biomolecules as physiological or pathophysiological responses. Mast cells densities are higher at acupoints [28-32]. There is evidence that acupuncture could also cause degranulation of mast calls directly through mechanical stress [30]. Thus, mast cells could be mediators of the effector functions of acupuncture action.

### Interstitial fluid flow along meridian channels

The whole interstitium is considered to be four times of the blood in volume. All extracellular fluids are in dynamic equilibrium with other fluid systems (like lymphatic fluid) of the body. The instantaneous interstitial fluid pressures Pi at different locations in the interstitium are not equal, depending on many factors, such as: (1) difference in protein and electrolyte concentrations between the blood and the interstitial fluid, (2) blood flow rates at the capillaries and venules, (3) pressures at the arterial and venous ends of these small vessels which vary in different organs, (4) the effective pumping activities of the lymphatic ducts [45,53].

The interstitial fluid pressures are different at different sites and different body positions [54-57]. As the interstitial fluid flows, a pressure acts on the adjacent connective tissues inside the interstitium and changes the shapes of these connective tissues. Such changes in pressure would in turn influence the flow speed and the value of Pi until a dynamic equilibrium is restored [58]. Like a stream flowing along a bank with stones of various sizes, not all the water molecules flow at the same speed; rather, the flow with the fastest speed emerges.

There is experimental evidence to show that interstitial fluid flows along the meridian channels as demonstrated by radioactive tracing studies [59-62]. On the other hand, other radioactive studies on animal and humans demonstrated that the tracks of flow were not along blood vessels [63-65]. The above experimental evidence of Chinese medicine meridian channel research, knowledge of physiology and basic physics suggest that the interstitial fluid is not stationary, but flows along certain parts of the body, which is a consequence of difference in Pi in different parts of the body. The lymphatic pumps are part, but not all the causes of fluid flow in IF.

Using biorheology techniques designed by Guyton *et al*. [66] and Levick [67], Zhang *et al*. [68,69] found that interstitial fluid flows with the smallest resistance existed along the longitudinal directions of animal bodies. The measured speed of flows is much slower than that of blood circulation.

Using magnetic resonance angiography and magnetic resonance imaging (MRI) techniques, Li *et al*. [70] demonstrated in humans that the six specific migration channels of interstitial fluid were not blood and lymphatic vessels. The flows followed the six Yin Chinese medicine meridian channels in the upper and lower limbs [70]. Thus, this is an important piece of evidence to support that some interstitial fluid flows along the six Yin meridian channels.

### Boundary tissue of the Chinese medicine meridian channels

Meridian channels embedded in the CTIF system provide specific paths of cell migration and interstitial fluid flows [28-32,59-65]. Such specific paths indicate that meridian channels should have tissue boundaries. While the tissue boundaries throughout meridian channels have not been fully elucidated, investigation into the sites of acupoints [2-8], which are hypothesized to be high efficient, specific functional sites along meridian channels, do provide information about the tissue boundaries of meridian channels near the acupoints. In particular, Yuan *et al*. [9] analyzed the images from digital data set derivable from slices of cadavers and found that 361 acupoints were located in five types of connective tissues: (a) dense connective tissue in the dermal reticular; (b) subcutaneous loose connective tissue; (c) intermuscular (loose) septa; (d) (loose) connective tissues embedding neuromuscular tracts; (e) (loose) connective tissues at the visceral hili and tunicae. Dang *et al*. [4]'s work indicated that 9 out of 11 of the acupoints of the lung meridian were on the periosteum, one on the perineurium of the (radial) nerve, another on the adventia of the (radial) artery. These findings suggest that the meridian channels are bound below by a layer of connective tissues of various types. On the other hand, longitudinally distributed lines with rich sympathetic substance (neurotransmitters) were found in the skin in animal studies by Liu *et al*. [71,72], suggesting that these lines with high nerve activities form the "upper boundary" of the Chinese medicine channels. As the acupoints along the meridian channels are proposed to be functional sites with high efficiency and specificity, they need to be: (1) near dense nerve structure (abundant nerve endings with polymodal and other receptors) and (2) dense vasculature, (3) near lymphatic vessels, (4) with interstitial fluid flowing through. Such characteristics are also supported by anatomical studies [7,73].

### The hypothesis for the anatomical structure of the Chinese medicine meridian channels and acupoints

Based on the modern concepts of biomedical science and recent advances in acupuncture research, the author puts forth the following hypothesis:

The Chinese medicine meridian channel system has a structure bounded by the skin where there are abundant nociceptive receptors of various types and bound below by another layer of connective tissue with flowing interstitial fluid (including proteins with surface charges and ions) as ground substance. The interstitial fluid in the meridian channel participates in the continuous redistribution of the interstitial fluid pressure Pi in the body during body movement. These extracellular channels provide favorable migratory tracks mainly due to durotaxis for mast cells, fibroblasts and other cells (including adult stem cells) which carry out a number of physiological functions like triggering neurogenic inflammation, vasotone homeostasis, wound repair, giving the organism the optimum chance of survival. Acupoints are functional sites along the meridian channels. Acupuncture applied to these sites could improve the efficiency of the above functions through mast cell degranulation with specificity.

## Conclusion

Based on modern concepts of biomedical science, an integrated picture of meridian channels structure and acupuncture in action at acupoints is hypothesized. The hypothesis, referred as the CFMDD hypothesis, defines the meridian structure embedded in the Connective Tissue Interstitial Fluid system with acupuncture in action explained by Mechanotransduction, cells Durotaxis and mast cell Degranulation.

## Competing interests

The author declares that they have no competing interests.
